# Small GTPase Arl6 controls RH30 rhabdomyosarcoma cell growth through ciliogenesis and Hedgehog signaling

**DOI:** 10.1186/s13578-016-0126-2

**Published:** 2016-12-12

**Authors:** Xiaotong Liu, Qiuhong Shen, Tingting Yu, Huijie Huang, Ziyu Zhang, Jie Ding, Ying Tang, Ning Xu, Shen Yue

**Affiliations:** 1 Department of Developmental Genetics, School of Basic Medical Sciences, Nanjing Medical University, Nanjing, 211166 Jiangsu People’s Republic of China; 2 Central Laboratory, The First People’s Hospital of Wujiang District, Suzhou, 215200 People’s Republic of China; 3 Department of Pathology, School of Basic Medical Sciences, Nanjing Medical University, Nanjing, 211166 Jiangsu People’s Republic of China

**Keywords:** Primary cilia, Hedgehog, Rhabdomyosarcoma

## Abstract

**Background:**

Rhabdomyosarcoma (RMS) originates from skeletal muscle precursors that fail to differentiate. Hedgehog (Hh) signaling and primary cilia contribute to the pathobiology of RMS.

**Results:**

Here we showed ADP ribosylation factor like GTPase 6 (ARL6) localizes at the base of primary cilium, controls ciliogenesis and Hh signaling. The transcription of Arl6 is dynamic during the differentiation of myoblasts, companying with the growth and elimination of primary cilia. Arl6 expression is significantly up regulated in cilia-dependent RMS cells and tissues. Knockdown of Arl6 inhibits proliferation and promotes apoptosis of RMS RH30 cells through defected ciliogenesis and reduced Hh activity.

**Conclusions:**

Taken together, the functions of Arl6 in ciliogenesis and Hh signaling suggest it as a potential RMS drug target.

**Electronic supplementary material:**

The online version of this article (doi:10.1186/s13578-016-0126-2) contains supplementary material, which is available to authorized users.

## Background

Rhabdomyosarcoma (RMS) is the most common soft tissue sarcoma in children, which originates from skeletal muscle precursors that fail to differentiate [1]. Current therapeutic approaches for RMS include multi-agent chemotherapy, radiation therapy and surgical resection, which have not resulted in significant improvement in outcome for patients with recurrence and/or metastasis [2]. Recent investigations realized that dysregulation in RAS pathway [3], insulin-like growth factor [4], p53 [5], AKT/mTOR [6] and Hedgehog (Hh) signaling [6–8] is associated with the pathobiology of RMS, which suggested those signaling pathways as potential RMS drug targets.

Abnormal assembly of primary cilia is recently reported in RMS [9]. Primary cilium is a microtubule-based cell protrusion present in every interphase cell, functions as a sensory organelle on the cell surface to detect a wide variety of stimuli [10, 11]. Many gene mutations cause defective ciliogenesis or ciliary transport, e.g. intraflagellar transport (IFT) genes, small GTPases and Bardet–Biedl syndrome (BBS) genes encoding proteins that form a complex termed the BBSome [12]. The detailed mechanisms are still not very clear. Vertebrate Hh signaling cascade is initiated by the binding of three Hh ligands, Sonic Hedgehog (Shh), Indian Hedgehog (Ihh) and Desert Hedgehog (Dhh) to the 12-pass transmembrane receptor Patched-1 (Ptch1). Hh signaling is absolutely dependent on primary cilia, and requires the IFT machinery for the transport and processing of its signaling components in primary cilia, including transmembrane receptors, Ptch1, Smoothened (Smo), and downstream GLI-Kruppel family of transcription factors Gli1-3 as well [12].

ADP ribosylation factor like GTPase 6 (Arl6), also named as BBS3, is a member of the Ras superfamily of small GTPase, which is mutated in human Bardet–Biedl syndrome [13, 14]. Arl6 is required for the ciliary localization of the BBSome complex [15], which is necessary for ciliary membrane biogenesis and sorting membrane proteins to cilia [15, 16]. Studies from *Caenorhabditis elegans* showed that Arl6 undergoes IFT [14]. Arl6 GTPase activity is required for Wnt signaling in cultured mammalian cells [17]. Recent studies from Arl6 knockout mice also showed that loss of Arl6 affects retrograde transport of Smo inside cilia [18]. However, the function of Arl6 in cilia-related RMS is still unknown.

Our data points to the role of Arl6 controlling RH30 RMS cell growth through ciliogenesis and Hedgehog signaling. During the in vitro differentiation of an established myoblast cell line, C2C12, we saw dynamic Arl6 expression and accompanying growth or elimination of primary cilia. Further more, Arl6 expression is significantly up-regulated in cilia-dependent RMS RH30 cells and tissues relative to normal skeletal muscles. RH30 cells with disrupted Arl6 expression show reduced ciliogenesis and crippled Hh activity, resulting in retarded cell growth as well as increased apoptosis.

## Methods

### Cell culture and plasmids construction

Wild type (WT) and Arl6 knockout mouse embryonic fibroblasts (MEFs) were gifts from Dr. Val C. Sheffield (University of Iowa, Iowa City, IA, USA), and were immortalized following NIH3T3 protocol. A mouse myoblast cell line C2C12 and human RMS cell lines RD and RH30 are from ATCC. RD and RH30 are derived from tumors of the embryonal and alveolar origin, respectively. MEFs, RD and RH30 cells were maintained in DMEM supplemented with 10% fetal bovine serum (FBS), 100 U/ml penicillin and 100 mg/ml streptomycin at 37 °C with 5% CO2. C2C12 cells were maintained with 15% FBS. Mouse Arl6 cDNA was obtained from MGC cDNA library (Open biosystems), and cloned into pRK5 vector with a red fluorescent protein (RFP) tag by PCR. The T31R and Q73L mutants of Arl6 were created using the Quickchange site-directed mutagenesis kit (Agilent Technologies).

The constructs expressing Arl6 short hairpin RNA (shArl6) (target sequence: GTCGAATTCCAATCTTGTT) and scramble control (shCon) were purchased from GeneChem (Shanghai, China). Briefly, the short hairpin RNAs were cloned into GV248, which has a hU6 promoter. The knock-down efficiency of shArl6 was validated by quantitative real-time polymerase chain reaction (Q-PCR). To generate stable cell lines, shArl6 or shCon plasmids were transfected in RD or RH30 cells using Fugene HD according to the manufacturer’s procedure (Promega, Madison, WI). Transfected cells were selected with 5 μg/ml puromycin for 2 weeks, and used for following experiments.

### RNA isolation and quantitative PCR

Total RNA was isolated from cultured cells using the RNAiso reagent (TaKaRa, Shiga, Japan), and reverse transcription was carried out using the PrimeScript RT reagent Kit (TaKaRa). Standard reverse transcription polymerase chain reaction (RT-PCR) was carried out with the following primers: mouse Gli1 (5′-TCCAGCTTGGATGAAGGACCTTGT-3′ and 5′-AGCATATCTGGCACGGAGCATGTA-3′) and mouse Hypoxanthine-guanine phosophoribosyltransferase (HPRT) (5′-TATGGACAGGACTGAAAGAC-3′ and 5′-TAATCCAGCAGGTCAGCAAA-3′). Q-PCR was carried out using the FastStart SYBR Green Master mix (Roche, Germany) on a LightCycler 96 System (Roche) with primers for mouse Arl6 (5′-CACCGTCGAATTCCAATCTTG-3′ and 5′-ATGGCGTCACTAG- CACAAATATG-3′), mouse Gli1 (5′-GCTTGGATGAAGGACCTTGTG-3′ and 5′-GCTGATCCAGCCTAAGGTTCTC-3′), mouse glyceraldehyde 3-phosphate dehydrogenase (GAPDH) (5′-AGGTCGG- TGTGAACGGATTTG-3′ and 5′-GGGGTCGTTGATGGCAACA-3′), human Arl6 (5′-GTGTCTCAGTTGCTGTGTTTAG-3′ and 5′-AGCCAGTCTACACCTT- CTTG-3′), human Gli1 (5′-TCCTCTGAGACGCCATGTTC-3′ and 5′-CAGACAGTCCTTCTGTCCCCA-3′), and human GAPDH (5′-ATCATCCCTGCCTCTACTGG-3′ and 5′-GTCAGGTCCACCACTGACAC-3′). Experiments were repeated at least three times, and samples were analyzed in triplicate.

### Western blot analysis

After transfection or treatment as described, cells were lysed in radioimmunoprecipitation assay buffer (RIPA buffer) (50 mM Tris-HCl, pH 7.4, 150 mM NaCl, 1% vol/vol NP-40, 0.25% wt/vol sodium deoxycholate, 0.25% wt/vol NaF, 10 mmol/l β-glycerolphosphate, 1 mM Na_3_VO_4_, 1 mM DTT, and 1× Roche cOmplete Protease Inhibitor Cocktail) for 1 h at 4 °C. The lysate was clarified by centrifugation for 1 h at 20,000×*g*. The protein concentration was determined using a bicinchoninic acid assay and equal amounts of total protein from each of the samples was supplemented with 6× SDS loading buffer, heated at 95 °C for 5 min, subjected to SDS-PAGE, followed by western blotting with indicated antibodies. The source for antibodies are: anti-Arl6 antibodies (Sigma-Aldrich, St. Louis, MO); anti-myogenin and anti-β-actin antibodies (Santa Cruz Biotechnology, Dallas, TX).

### Immunohistochemical staining

Ptch1^+/−^ mice (Jackson Laboratory, Bar Harbor, ME) were outcrossed onto the CD1 background for spontaneous RMS development. RMS and normal muscle tissues were dissected from gastrocnemius of hindlimbs of Ptch1^+/−^ mice. Immunohistochemistry of tissue sections with antibodies to Gli1 (Cell Signaling Technologies, Danvers, MA) and Arl6 (Sigma-Aldrich) was performed using Polink-2 plus^®^ Polymer HRP Detection System (ZSGB-BIO, China). Nuclei were counterstained with hematoxylin. Sections were visualized under 40× magnification using Leica DMI 3000B.

### Immunofluorescent staining and confocal microscopy

Approximately 5 × 10^4^ cells per well were seeded in Millicell EZ slides and cultured for 24 h. The cells were transfected, allowed to recover for 24–36 h. MEFs were treated with 0.5% FBS for 24 h to induce cilia growth. C2C12 or RMS cell lines were treated with 2% horse serum (HS) for 48 h to induce cilia growth. Cells were fixed with 4% paraformaldehyde for 10 min at 4 °C, and standard procedures for immunostaining were followed. Primary cilia were stained with mouse anti-acetylated tubulin (1:2000, Sigma-Aldrich) and corresponding Alexa-coupled secondary antibodies (1:200, Life Technologies). Tissue sections were immunostained with anti-acetylated tubulin. Confocal images were acquired on a Carl Zeiss LSM710 microscope system using Z-stack module of ZEN2011 program.

### MTT assay

To measure cell growth, RD and RH30 cells stably expressing shArl6 or control shRNA were seeded at 3000 cells per well in 96-well plates. For 5 days, cells were incubated with 100 μl MTT (3-(4, 5-dimethylthiazol-2-yl)-2, 5-diphenyl-tetrazoliumbromide, 5 mg/ml) for 4 h at 37 °C. Following MTT incubation, 100 μl DMSO was added to dissolve the crystals and the absorbance at 490 nm was measured using a microplate absorbance reader (Tecan, Austria). Each assay was performed in triplicates and repeated three times independently.

### EdU incorporation

To measure cell proliferation, RD and RH30 cells stably expressed expressing shArl6 or control shRNA were seeded at 2 × 10^4^ cells per well in 24-well plates and cultured for 48 h. Cells were incubated with 10 μM EdU for 2 h before the end of the culture period. Following culture, cells were, fixed with 3.7% formaldehyde, and then detected with Click-iT EdU Imaging Kit (Life Technologies, CA) according to the manufacturer’s procedure (Life Technologies, CA). The plates were visualized under 20× magnification using Leica DMI 3000B with Leica DFC490 Digital Camera. Quantification of percentage of EdU-positive cells was performed using the ImageJ program.

### Cell cycle and apoptosis assays

Subconfluent RH30 stable cells were synchronized with 0.5% FBS for 24 h, then rescued with 10% FBS for 12 h before cell cycle analysis. To assess cell cycle properties, cells were collected and incubated with 100 μg/ml propidium iodide and 0.5 μg/ml RNase A for 30 min at the room temperature before subjecting to FACS analysis. To assess apoptosis, stable cells were cultured with 0.2% FBS for 72 h to induce apoptosis. The apoptotic cells were analyzed using the Annexin V: FITC apoptosis detection kit according to the manufacturer’s instruction (BD, USA).

### Generation of Arl6-knockout RH30 cells

To make CRISPR/Cas9 constructs, a pair of single guide RNAs (sgRNAs) targeted human Arl6 were designed and cloned into pX330 vector (from Addgene) [19]. Arl6 sgRNAs target sequences are 5′-TGCCACTATTATCTAGCCCAAGG-3′ and 5′-GACAGACTTTCAGTCTTGCTTGG-3′. To generate Arl6 knockout cells, RH30 cells were co-transfected with CRISPR/Cas9 plasmids and the ones encoding puromycin selection marker. 48 h after transfection, transfection-positive cells were selected using puromycin. After 5 days of selection, survived RH30 cells were seeded at 30 cells per well in 96-well plates and screened for Arl6 levels by western blotting.

### Statistical analysis

Statistical analyses were performed in the GraphPad Prism 5.0 environment. Comparisons between indicated groups were performed using independent-samples *t* test. P values less than 0.05 were considered statistically significant. *p < 0.05, **p < 0.01, and ***p < 0.001.

## Results

### Arl6 controls ciliogenesis and Hh signaling from the basal body to the primary cilium

**Fig. 1:**
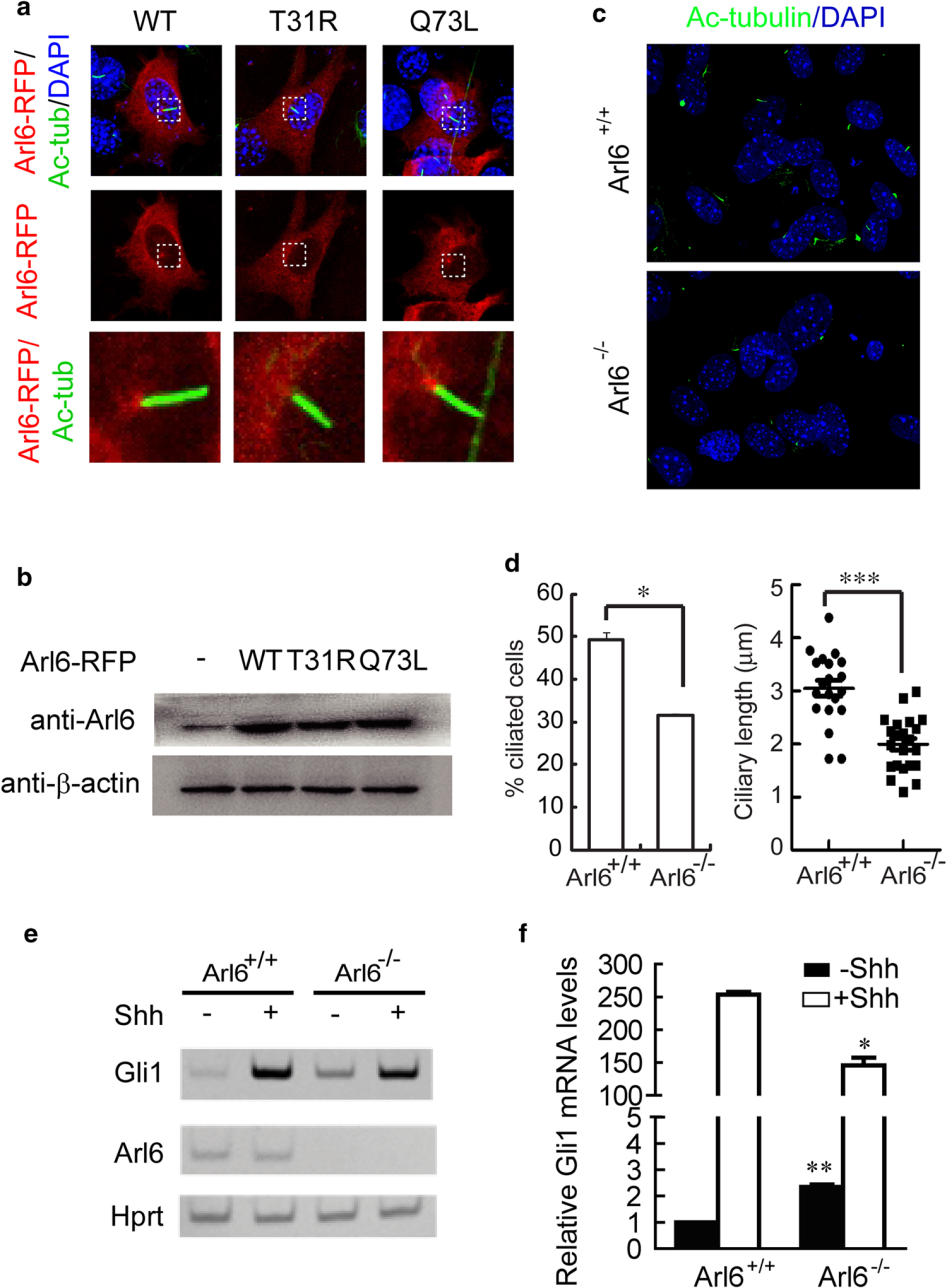
Arl6 regulates ciliogenesis and Hedgehog signaling transduction at the base of primary cilium. **a** Confocal images showing distribution of exogenously expressed Arl6-RFP (*red*) or its T31R, Q73L mutants in MEFs. Arl6-RFP and its Q73L mutant accumulated in basal body of primary cilium (*green*), but T31R did not. **b** Western blot showing the same expression level of Arl6-RFP and its mutants in MEFs. β-actin was used as a loading control. **c** Representative confocal images of anti-acetylated tubulin (*green*) showing the lack of primary cilium in stable Arl6^−/−^ MEFs compared to WT. Nucleus are stained with DAPI (*blue*). **d** Quantitation of percentage (*left*) and ciliary length (*right*) of ciliated cells from **c**. **e** RT-PCR detection of Gli1, Arl6 in WT and Arl6^−/−^ MEFs stimulated with Shh conditional medium. HPRT mRNA was used as an internal control. A representative gel image is shown here. The relative intensity of the bands is shown in Additional file 1: Figure S1. **f** Q-PCR quantification of fold induction of Gli1 mRNA from an experiment as in **e**. Fold induction was calculated using Gli1 mRNA level normalized against that of HPRT for even loading and then against the normalized Gli1 mRNA level from WT cells without Shh treatment. The results represent the mean ± standard error of the mean (SEM) of three independent experiments *P < 0.05, ***P < 0.001 vs. Arl6^+/+^ control

Arl6 is a small GTPase critical for ciliary transport of BBSome [15, 20]. To determine the ciliary localization of Arl6, we ectopically expressed mouse Arl6 tagged with RFP (Arl6-RFP) in MEF cells, then serum-starved those MEF cells with 0.5% FBS for 24 h to induce ciliogenesis. The Arl6-RFP predominantly distributed in cytoplasm but not nucleus. With the anti-acetylated tubulin staining of primary cilia, we also found accumulation of Arl6-RFP at the base of cilia (Fig. 1a). However we didn’t find localization of Arl6 in primary cilia as it reported before [15]. The failed modification of ectopically expressed Arl6 is one possible reason for its failed transport into cilia. To access whether the GTP binding is necessary for localization of Arl6 at ciliary base, we ectopically expressed Arl6 mutants T31R and Q73L in MEFs as well. T31R is a dominant-negative mutant appears exclusively bound to GDP, while Q73L is a constitutively active mutant appears strictly bound to GTP. Under confocal microscope, we found that Q73L mutant accumulated at the base of cilia as WT did, while T31R didn’t, although they expressed at similar levels (Fig. 1a, b). It suggested that GTP-bound Arl6 functions at the base of primary cilia.

It was reported that Arl6 functions at or near the ciliary gate to modulate mammalian ciliary assembly or disassembly and ciliary signaling, for example Wnt Signaling [17]. To understand the function of Arl6 at the base, we immortalized Arl6^−/−^ MEFs derived from E13.5 Arl6 knockout mice [18]. Our immortalized Arl6^−/−^ MEFs showed reduced numbers and length of primary cilia (Fig. 1c, d), although ciliary staining was normal in tissue sections from kidney, eye, and pancreas of Arl6^−/−^ mice [18]. Vertebrate Hh signaling absolutely requires IFT [4]. To assess the role of Arl6 in Hh signaling, we quantified the transcriptional responses of endogenous Gli1 by RT-PCR and Q-PCR in Arl6^−/−^ MEFs, and found that lack of Arl6 led to marked curtailment of Gli1 activation (Fig. 1e, f; Additional file 1: Figure S1). Knockdown of Arl6 leads to a 42.5% decrease of Hh pathway activation. This result is consistent with the disruption of ciliogenesis in Arl6^−/−^ MEFs. Taken together, the above results show that Arl6 is required for ciliogenesis and vertebrate Hh signaling.

### Arl6 is expressed dynamically during muscle differentiation

**Fig. 2:**
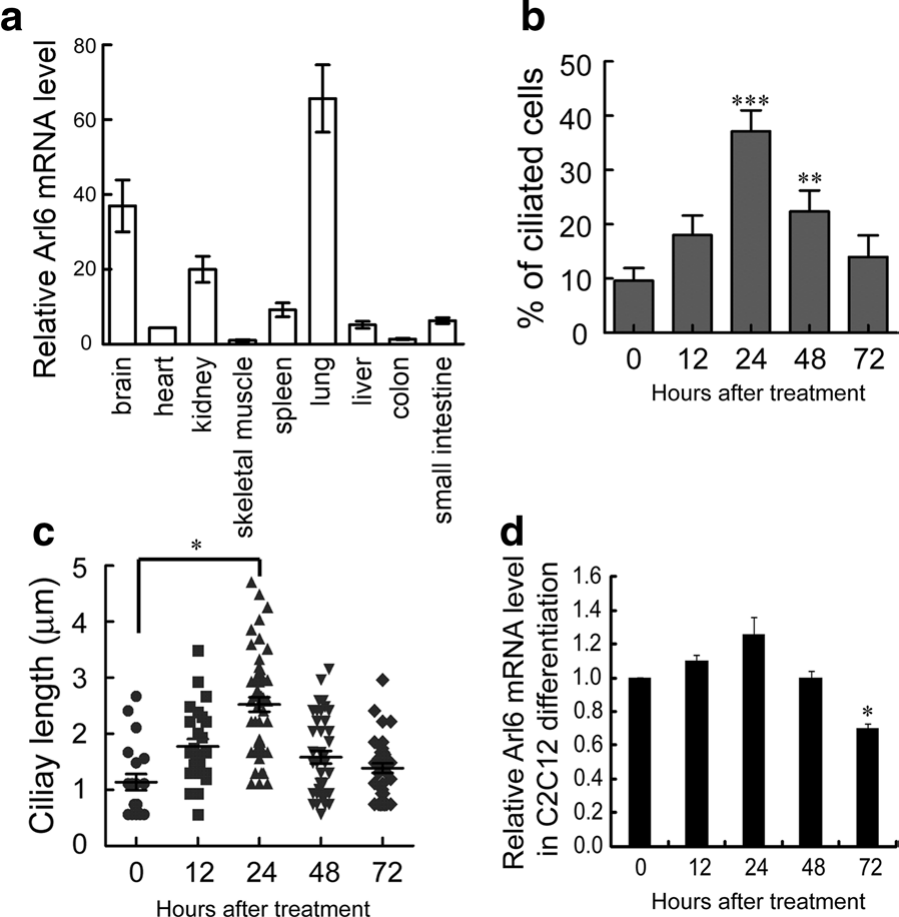
Primary cilia and Arl6 are dynamically regulated during myogenic differentiation of C2C12 cells. **a** Q-PCR quantification of Arl6 in mouse tissues. The results represent the mean ± SEM of three independent experiments. Quantitation of percentage (**b**) and ciliary length (**c**) of ciliated C2C12 cells during myogenic differentiation. Cells were incubated in differentiation medium containing 2% HS for 3 days. The *error bars* denote SEM. Representative confocal images are shown in Additional file 2: Figure S2. **d** Q-PCR analysis of mRNA level of Arl6 during differentiation. The data represents the mean ± SEM of three independent experiments. *P < 0.05, **P < 0.01, and ***P < 0.001 vs. 0 h

To address the function of Arl6, we first examined the expression pattern in adult mouse tissues by Q-PCR. High expression of Arl6 was found in brain, kidney and lung, which have plenty of ciliated cells (Fig. 2a). In contrast, little expression was found in heart, skeletal muscle and colon, which are muscle tissues without cilia (Fig. 2a). Arl6 expression pattern in mouse is quite similar as that in human according to records from *The Human Protein Atlas* database (http://www.proteinatlas.org/ENSG00000113966-ARL6/tissue). It suggested that Arl6 functions in ciliogenesis or ciliary transport in mammals.

Although primary cilia are missing in mature muscle, they grow in myoblasts. Recent study reported that assembly and disassembly of primary cilia are controlled during the differentiation of myoblasts [9]. To understand whether Arl6 contributes to ciliogenesis during muscle differentiation, we induced the differentiation of C2C12 myoblasts with 2% HS for 3 days, and analyzed ciliary formation and Arl6 expression at different time points. The increased expression of differentiation marker Myogenin over time indicates C2C12 cells differentiated well as we expected (Additional file 2: Figure S2B). We found that primary cilia were assembled during the early stage of myogenic differentiation (0–24 h after HS treatment), subsequently disappeared during the later period (24–72 h after HS treatment) through immunofluorescent staining of acetylated tubulin (Additional file 2: Figure S2A). The numbers and length of cilia reached the peak at 24 h time point (Fig. 2b, c). This result is consistent with that of primary mouse myoblasts [9]. Interestingly, the transcription of Arl6 was also dynamically regulated during the differentiation of C2C12 cells. The mRNA level of Arl6 was also at the peak at 24 h time point according to Q-PCR analysis (Fig. 2d). So the regulation of Arl6 expression synchronized with that of ciliogenesis. These results suggested that Arl6 participates ciliogenesis during muscle differentiation.

### Up-regulation of Arl6 is related to RMS

**Fig. 3:**
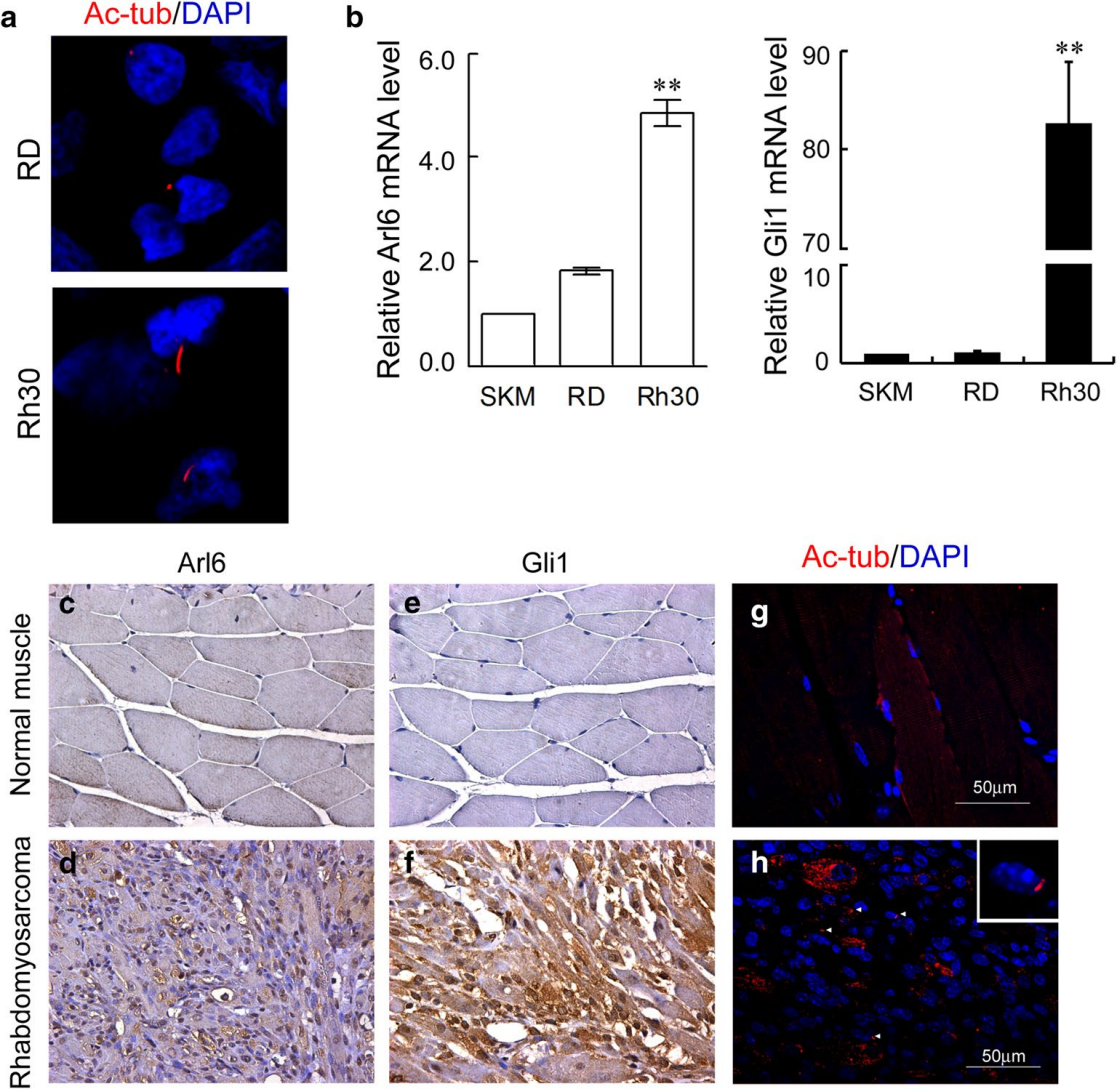
Arl6 and Hedgehog pathway is deregulated in ciliated RMS. **a** Representative images of anti-acetylated tubulin (*red*) staining in RD and RH30 cell lines. RMS cells were treated with 2% HS for 48 h before fixation for ciliogenesis. **b** Q-PCR quantification of Arl6 (*left*), Gli1 (*right*) in RD and RH30 cells, as well as in normal skeletal muscles (SKM). The data represents the mean ± SEM of three independent experiments. **P < 0.01 vs. RD. IHC staining of Arl6 (**c**, **d**) or Gli1 (**e**, **f**) in normal muscle and RMS from Ptch1^+/−^ mouse. **g**, **h** Staining of primary cilia (*red*) and nuclei (*blue*) in the same tissues from **c**–**f**

It is reported that ciliogenesis is deregulated in RMS, which is derived from myogenic precursors [9]. We found that primary cilia were observed only in RH30 cells but not RD cells after 48 h treatment with 2% HS (Fig. 3a), which is consistent with the reports from other groups [7, 9]. Interestingly, mRNA level of Arl6 was increased dramatically in RH30 cells compared with SKM and RD cells (Fig. 3b *left*). Meanwhile our Q-PCR also showed dramatic increase of Gli1 in RH30 cells as well (Fig. 3b *right*). It is well known that aberrant Hh activity initiates or promotes RMS tumorigenesis [21, 22]. Previous studies have shown that RMS formation in the patients with Ptch1 mutation and Ptch1^+/−^ mice as well [23]. So we are curious whether Arl6 is involved in Hh-associated RMS in vivo. To answer this question, we separated RMS tumor tissues and normal gastrocnemius tissues from same Ptch1^+/−^ mouse for IHC staining. The IHC results showed a robust Arl6 and Gli1 expression in RMS tissues (Fig. 3d, f). In contrast, in the gastrocnemius control, Arl6 and Gli1 expression was barely detectable (Fig. 3c, e). The typical primary cilia were seen in RMS sections but not in normal skeletal muscle control (Fig. 3g, h). These in vitro and in vivo evidences suggested that up-regulation of Arl6 caused abnormal assembly of primary cilia and aberrant activation of Hh signaling in RMS.

### Arl6 promotes RH30 cell growth and inhibits ciliogenesis in RH30 cells

**Fig. 4:**
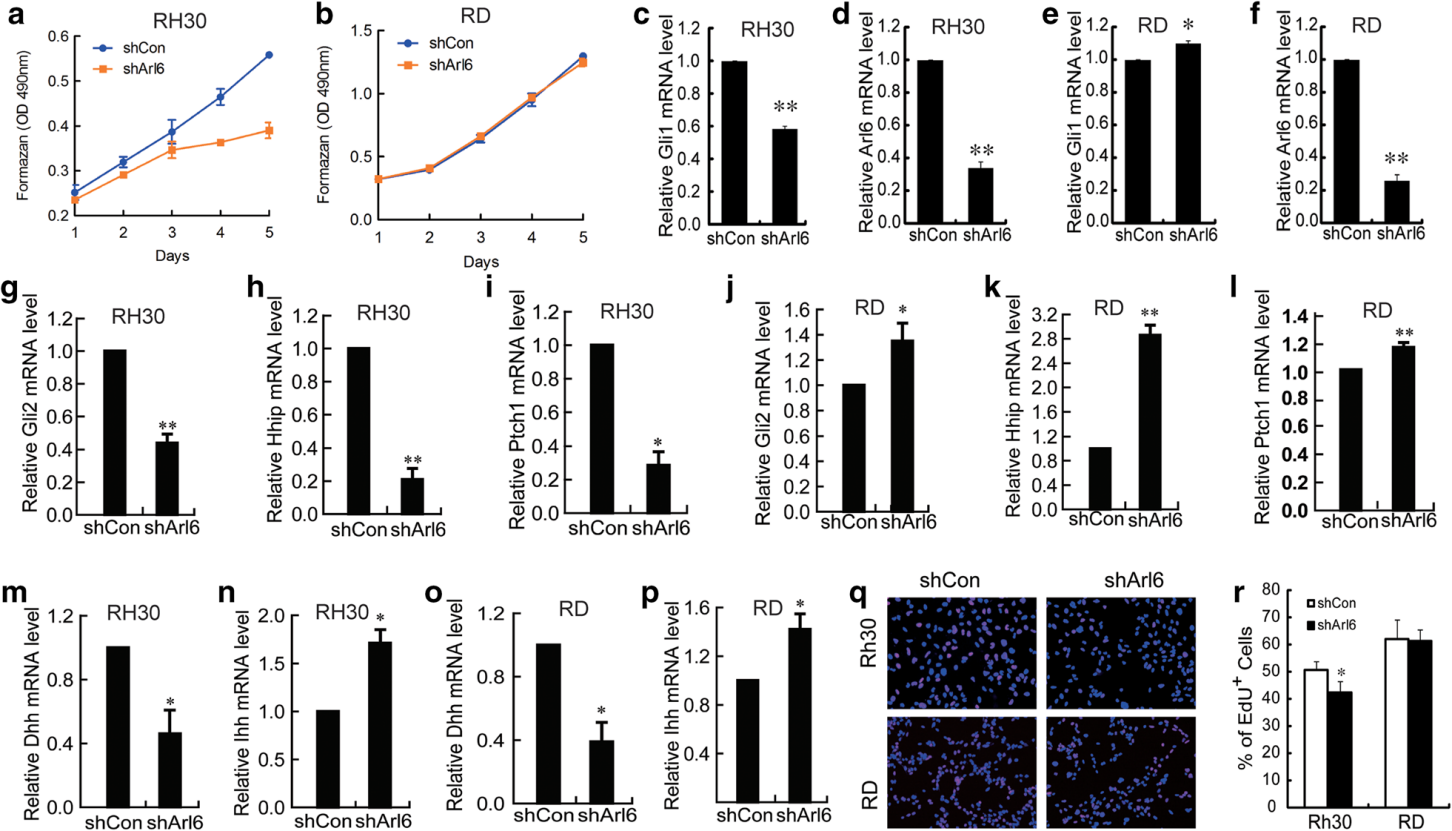
Knockdown of Arl6 suppresses cell growth of RH30. MTT assays for RH30 (**a**) and RD (**b**) cells stably expressing shArl6 or nonsense control. Q-PCR detection of Gli1 mRNA levels in RH30 (**c**) and RD (**e**) stable cells. The knockdown efficiency of shArl6 in RH30 (**d**) and RD (**f**) cells were also detected by Q-PCR. Q-PCR detection of Hh target genes Gli2, Hhip and Ptch1 mRNA levels in RH30 (**g**, **h**, **i**) and RD (**j**, **k**, **l**) stable cells. Q-PCR detection of Hh ligands Dhh and Ihh mRNA levels in RH30 (**m**, **n**) and RD (**o**, **p**) stable cells. **q** Representative fluorescence images and **r** percentage quantification of EdU incorporation assays of RH30 and RD stable cells. The cells were assayed in 24-well plates. The data was obtained in triplicate. Data in **a**–**p** and **r** are presented as mean ± SEM of three independent experiments. The *error bars* denote SEM. *P < 0.05 and **P < 0.01 vs. shCon

Having found the correlation between Arl6 expression, ciliogenesis and Hh signaling, we sought to address the physiological relevance of Arl6 regulation of ciliogenesis in RMS tumorigenesis. For this purpose, we generated RD and RH30 stable cell lines expressing shArl6 or shCon, and measured their proliferation. Relative to those expressing shCon, RH30 cells expressing shArl6 grew much slower and had reduced rate of metabolism as evident by quantification using MTT (Fig. 4a). Companying with the decrease of Arl6 expression, the mRNA level of Gli1 was significantly decreased in RH30 cells expressing shArl6 (Fig. 4c, d). However, knockdown of Arl6 didn’t reduce the rate of metabolism and Gli1 expression in RD cells (Fig. 4b, e, f). The mRNA levels of Hh target genes Ptch1, Hhip, and Gli2 were significantly decreased in RH30 but not RD cells knockdown of Arl6 (Fig. 4g–l). The decreased expression of Dhh and increased Ihh was found in RH30 and RD cells knockdown of Arl6 (Fig. 4m–p). Shh was undetectable. This result suggested that knockdown of Arl6 suppress Hh pathway activity through retarding the signaling transduction not ligands expression in RH30 cells. We then examined the role of Arl6 in sustaining the proliferation of RD and RH30 cells. Consistent with the decrease of rate of metabolism, the proliferation of RH30 cells was blocked by knockdown of Arl6 (Fig. 4q, r) according to the EdU incorporation results. Taken together, these results indicate that knockdown of Arl6 inhibits the growth and Hh activity of RH30 cells.

**Fig. 5:**
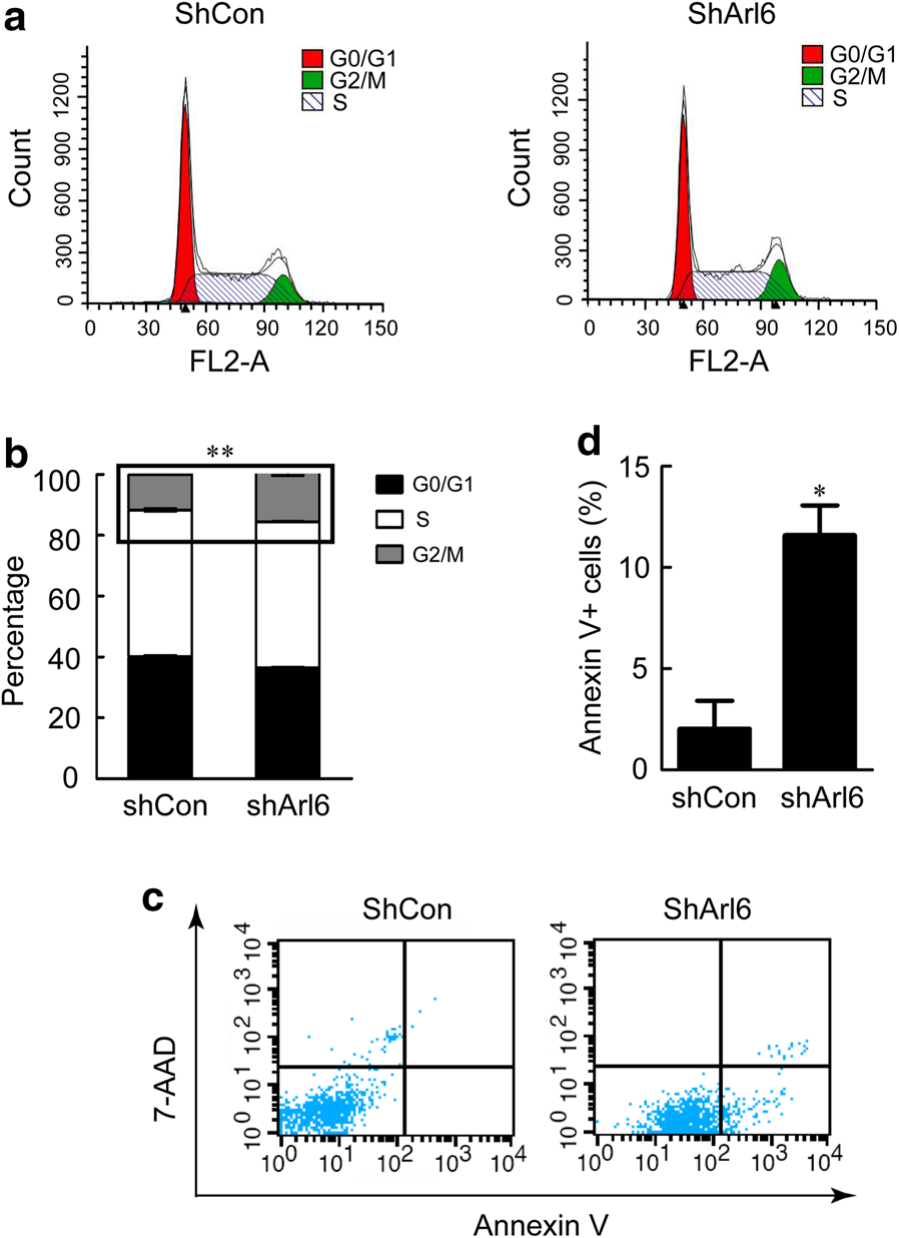
Knockdown of Arl6 induced cell cycle arrest and apoptosis of RH30 cells. **a** FACS analysis of the cell cycle profile in RH30 cells stably expressed shArl6 or nonsense control. 10,000 cells were analyzed in each sample. Quantitation of cell cycle distribution of RH30 cells from experiment (**a**) was shown in **b**. For apoptosis assays (**c**), the cells were maintained in DMEM supplemented with 0.2% FBS and processed for apoptosis assay 2 days later by Annexin V/PI double staining and FACS analysis. **d** Apoptotic rate was shown as the percentage of Annexin V + cells. The data represents the mean ± SEM of three independent experiments *P < 0.05, **P < 0.01 vs. shCon

To understand the mechanisms underlying the growth suppression effect of Arl6 knockdown, the cell cycle distribution of RH30 cells was analyzed. Knockdown of Arl6 induced G2/M phase arrest in RH30 cells. The fraction of RH30 cells in the G2/M phase increased from around 11.6% in the shCon to around 15.4% in the shArl6 treatment groups (Fig. 5a, b). The G2/M arrest was largely at the expense of the G0/G1 phase.

To further characterize the tumor suppression properties of Arl6 knockdown, we examined the ability of RH30 cells to undergo apoptosis when stably expressing shArl6. Apoptosis was induced by maintaining the cells in DMEM supplemented with 0.2% FBS for 24 h and quantified by using Annexin V and propidium iodide double staining. Flow cytometry analyses indicated that only around 1.5% of RH30 cells expressing shCon became Annexin V positive, but this percentage increased to 11.6% in the cells expressing shArl6 after serum depletion (Fig. 5c, d). This result indicated that knockdown of Arl6 promotes the apoptosis of RH30 cells, and suggested that cell cycle arrest at G2/M phase is involved in apoptosis induction.

**Fig. 6:**
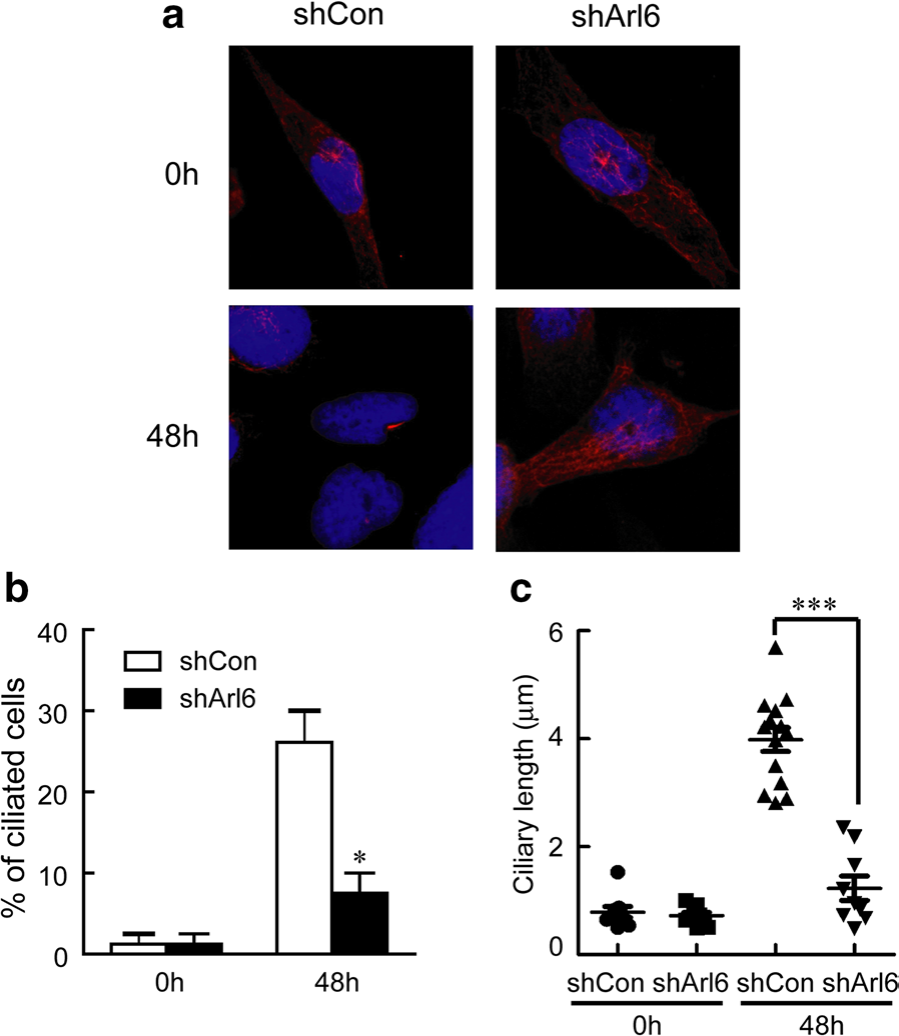
Arl6 is required for ciliogenesis of RH30 RMS cells. **a** Representative confocal images showing primary cilium in RH30 cells stably expressing shArl6 or shCon with anti-acetylated tubulin (*red*) staining. RMS cells were treated with 2% HS for 48 h to induce ciliogenesis. Quantitation of percentage (**b**) and ciliary length (**c**) of ciliated cells shown in **a**. The *bars* denote SEM. *P < 0.05, ***P < 0.01 vs. shCon

Since knockdown of Arl6 only obstructed the growth of RH30 cells but not RD cells, we expected the effect of shArl6 is related to the interruption of ciliogenesis in RH30 cells. So we stained the primary cilia in those RH30 stable cells, and found that RH30 cells stably expressing shArl6 could not form cilia even after 48 h treatment with 2% HS (Fig. 6a). As shown in Fig. 6b, around 26.1% RH30 cells stably expressing shCon showed typical structures of primary cilia, while only 7.5% RH30 cells stably expressing shArl6 had primary cilia after the induction of ciliogenesis with HS. Those data suggested that knockdown of Arl6 suppressed RH30 cell proliferation through interrupted ciliogenesis.

To identify the role of cilia assembly in RMS cell proliferation, we tried to knockdown IFT88 in RH30 cells (Additional file 3: Figure S3A). IFT88 is a well-known central component of the IFT complex B, essential for cilia assembly. Knockdown of IFT88 blocked cilia assembly, retarded Hh signaling, and suppressed cell proliferation (Additional file 3: Figure S3). The siIFT88 data identified that blocking cilia assembly suppresses Hh signaling and cell proliferation in RH30 cells.

To further investigate the functions of Arl6 in ciliogenesis and cell proliferation of RH30 cells, we used the CRISPR/Cas9 genome editing technology to knockout Arl6. Western blotting showed that the expression of Arl6 was significantly decreased in RH30 cells edited by Arl6 CRISPR, indicated Arl6 was knockout in most cells of the selected individual clone (Additional file 4: Figure S4A). The ciliogenesis and cell proliferation was also suppressed in edited RH30 cells when compared with that in untransfected cells (Additional file 4: Figure S4B–D). The data again indicated that Arl6 promotes cell growth and ciliogenesis in RH30 cells.

### Hh signal induces the transcription of Arl6

**Fig. 7:**
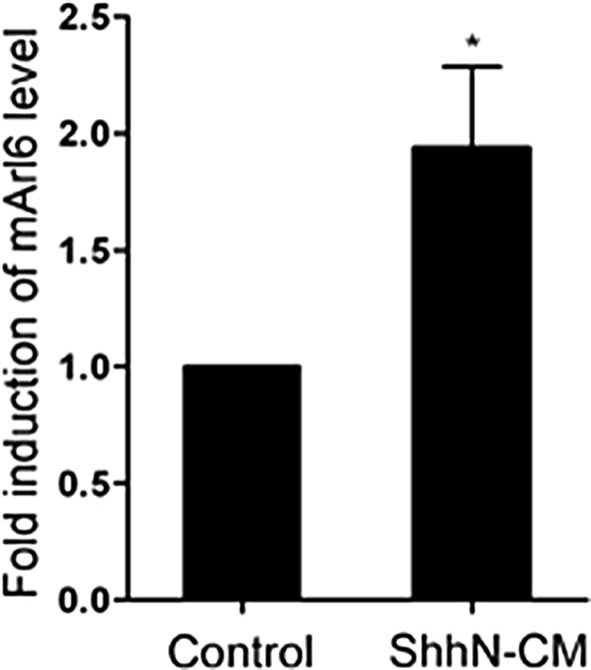
The transcription of Arl6 is induced by Hh signal. Q-PCR quantification of Arl6 in MEFs treated with Shh-conditioned medium harvested from the supernatant of HEK293 cells transiently transfected with a Shh-encoding plasmid. The results represent the mean ± SEM of three independent experiments. *P < 0.05 vs. control

Until now, we understand that Arl6 is involved in ciliogenesis and Hh signaling in myoblast differentiation and RMS tumorigenesis. However, the up-regulation of Arl6 in RMS tissues from Ptch1^+/−^ mouse is still not explained. To answer this question, we incubated wild type MEFs with Shh conditioned medium. As shown in Fig. 7, the Shh ligand induced Arl6 transcription in MEFs, indicating that Arl6 may be the direct or indirect target gene of Hh pathway. It suggested a positive feedback loop between Hh signal and Arl6.

## Discussion

RMS is the most common pediatric soft-tissue sarcoma that displays defective myogenic differentiation. Understanding the mechanisms in RMS development is important to invent innovative treatment strategies. In this study, we investigated the role of a small GTPase Arl6 in regulating RMS cell growth. Our research indicates that Arl6 is an oncogene in RMS cells. Arl6 expression is significantly up regulated in cilia-dependent human RMS cells RH30 and mouse RMS tissues. Knockdown of Arl6 obstructs the proliferation and promotes apoptosis of RH30 through defected ciliogenesis and reduced Hh activity.

Ciliogenesis is required for the differentiation of diverse cell types, for example, cardiomyocyte [24], adipocyte [25], myocyte [9, 26], osteocyte [27], and mesenchymal stem cells as well [28]. Assembly and disassembly of primary cilia are dynamical during the myogenic development. Primary cilia were assembled during the early stage of myogenic differentiation, subsequently disappeared during the later period. Interruption of cilia assembly with knockdown of IFT genes enhances the cell proliferation of myoblasts and abolishes the differentiation [9]. However, primary cilia assembly and disassembly are deregulated in RMS that originates from poorly differentiated myoblasts. Some kinds of RMS cells (A204, RH30, and Rh36) are ciliated [9]. We found that interruption of cilia assembly with knockdown of Arl6 obstructed cell proliferation and promoted apoptosis in ciliated RH30 cells. It suggests that the ciliary signaling transductions regulating cell cycle are different between myoblasts and RMS cells.

Arl6 is a ciliary protein, which accumulates at the base of primary cilia in our in vitro cultured MEFs and ciliated murine inner medullary collecting duct (IMCD3) cells [17], as well as in *C. elegans* [14]. This localization of Arl6 suggests its function in ciliogenesis and ciliary trafficking. However, recent study in Arl6 knockout mice indicated that Arl6 is dispensable in ciliogenesis, since the cilia morphology was completely normal in tissue sections from kidney, eye, and pancreas of Arl6^−/−^ mice [18]. There are numerous mice studies show that the phenotypic effects of single gene knockout are influenced by the genetic background [29]. It is possible that genetic redundancy masks some essential functions of Arl6 in those knockout mice. It is also possible that some spontaneous mutations during immortalization and in vitro short-term serum starvation potentiate cilia defect in Arl6^−/−^ MEFs. In our study, primary cilia numbers were significantly decreased in in vitro cultured Arl6^−/−^ MEFs compared with WT cells. As a small GTPase, the localization of Arl6 in primary cilia depends on the binding of GTP. T31R, a mutant of Arl6 reported in BBS3 pathology, appears strictly bound to GDP, although WT Arl6 is primarily bound to GTP. T31R mutant was on longer accumulated in basal body of primary cilia in our experiment. Interestingly, it was reported that expression of T31R leaded to stunted cilia in IMCD3 cells [30]. This result is similar to the loss of ciliogenesis in our MEFs and RH30 cells which lost Arl6. Those findings suggest that Arl6 regulates ciliogenesis in some situations.

Arl6 is critical for recruiting the polymerized BBSome to lipid vesicles as a coat and for targeting the BBSome to cilia but not the assembly of it [15]. Normal assembly and ciliary trafficking of BBSome is required for ciliary Hh signaling. Loss of BBS7, a subunit of BBSome, results in accumulation of Smoothened and Patched1 in cilia and decreases Hh response [31]. Loss of Arl6 affects the retrograde transport of Smoothened in cilia and leads to decreases in ciliary Hh signaling [18]. Our quantitative PCR for Gli1 from immortalized Arl6^−/−^ MEFs showed a slight increase without Hh and a significant decrease when stimulated with Hh compared with WT MEFs. The deregulation of Hh pathway activation is due to the deregulated transport at ciliary base lack of Arl6. Taken together with the function of Arl6 in ciliogenesis, Arl6 is critical to ciliary Hh signaling. In ciliated RMS cells and tissues, the Hh signaling was aberrantly activated accompanying with the high Arl6 expression. Aberrant activation of Hh signaling leads to oncogenic consequences involving deregulation of cell cycle and cell proliferation not only in RMS but also in other tumors as well [32, 33]. Hence, knockdown of Arl6 could inhibit the proliferation and promote of apoptosis of RMS cells through blockage of ciliary hedgehog signaling. Our study also showed that Hh signal could induce the transcription of Arl6. So there may be a positive feedback between Hh signaling and Arl6 to accelerate aberrant development of RMS.

## Conclusions

In conclusion, our data provides evidence that the small GTPase Arl6 contributes to RMS cell growth due to its function in ciliogenesis and ciliary Hh signaling. While Hh signal induces the transcription of Arl6 to form a positive feedback loop to maintain the pathway activation. The function of Arl6 may be dependent on its binding of GTP, since of the GTP-bound Arl6 localized at the basal body of primary cilia. The data supports that targeting Arl6 may be successful in the treatment of ciliated RMS. More research is needed to develop the compounds inhibiting the expression or GTP-binding of Arl6 for tumor therapy.

## Additional files

**Additional file 1: Figure S1.** Knockout of Arl6 reduced Hedgehog signaling activity in MEFs. Detection of Gli1 in Arl6 knockout MEFs by RT-PCR (Fig. 1e). The relative intensity of the bands is shown by semi-quantified analysis with the software Image J. The results represent the mean ± SEM of three independent experiments. **P < 0.01 compared with Arl6^+/+^.

**Additional file 2: Figure S2.** Cilia assemble and dissemble in myoblast differentiation. (A) Representative confocal images showing the anti-acetylated tubulin (green) staining of primary cilia on C2C12 during differentiation. (B) Western blot analysis of Myogenin levels during differentiation. β-actin was used as a loading control.

**Additional file 3: Figure S3.** Knockdown of IFT88 suppresses cell growth of RH30. (A) The knockdown efficiency of IFT88 siRNA in RH30 was detected by Q-PCR. (B-D) Q-PCR detection of Gli1, Ptch1 and Hhip mRNA levels in RH30 cells knockdown of IFT88. (E) Representative confocal images and (F) percentage quantitation of primary cilia in RH30 cells expressing siIFT88 or siCon. (G) MTT assays for RH30 cells expressing siIFT88 or siCon. The results represent the mean ± SEM of three independent experiments. ***P < 0.001 vs. siCon.

**Additional file 4: Figure S4.** Arl6 CRISPR suppresses cell growth of RH30. (A) Western blot evaluated Arl6 protein levels in selected individual RH30 clones edited by Arl6 CRISPR. (B) Representative confocal images and (C) percentage quantitation of primary cilia in RH30 edited cells. The results represent the mean ± SEM of three independent experiments. **P < 0.01 vs. WT (D) MTT assays for RH30 edited cells.

## Abbreviations

RMS: rhabdomyosarcoma
Hh: Hedgehog
ARL6: ADP ribosylation factor like GTPase 6
IFT: intraflagellar transport
BBS: Bardet–Biedl syndrome
Shh: Sonic Hedgehog
Ihh: Indian Hedgehog
Dhh: Desert Hedgehog
Ptch1: patched1
Smo: smoothened
WT: wild type
MEF: mouse embryonic fibroblast
FBS: fetal bovine serum
RFP: red fluorescent protein
shArl6: Arl6 short hairpin RNA
shCon: shRNA scramble control
Q-PCR: quantitative real-time polymerase chain reaction
RT-PCR: reverse transcription polymerase chain reaction
HPRT: hypoxanthine–guanine phosophoribosyltransferase
GAPDH: glyceraldehyde 3-phosphate dehydrogenase
RIPA: radioimmunoprecipitation assay
MTT: 3-(4,5-dimethylthiazol-2-yl)-2,5-diphenyl tetrazolium bromide
HS: horse serum
sgRNAs: single guide RNAs
Arl6-RFP: Arl6 tagged with RFP
IMCD3: murine inner medullary collecting duct
SEM: standard error of the mean
