# Supplementary figures and images for: Small GTPase Arl6 controls RH30 rhabdomyosarcoma cell growth through ciliogenesis and Hedgehog signaling
Source: Cell Biosci. 2016 Dec 12;6:61. doi: 10.1186/s13578-016-0126-2 (PMC5154108; doi:10.1186/s13578-016-0126-2)

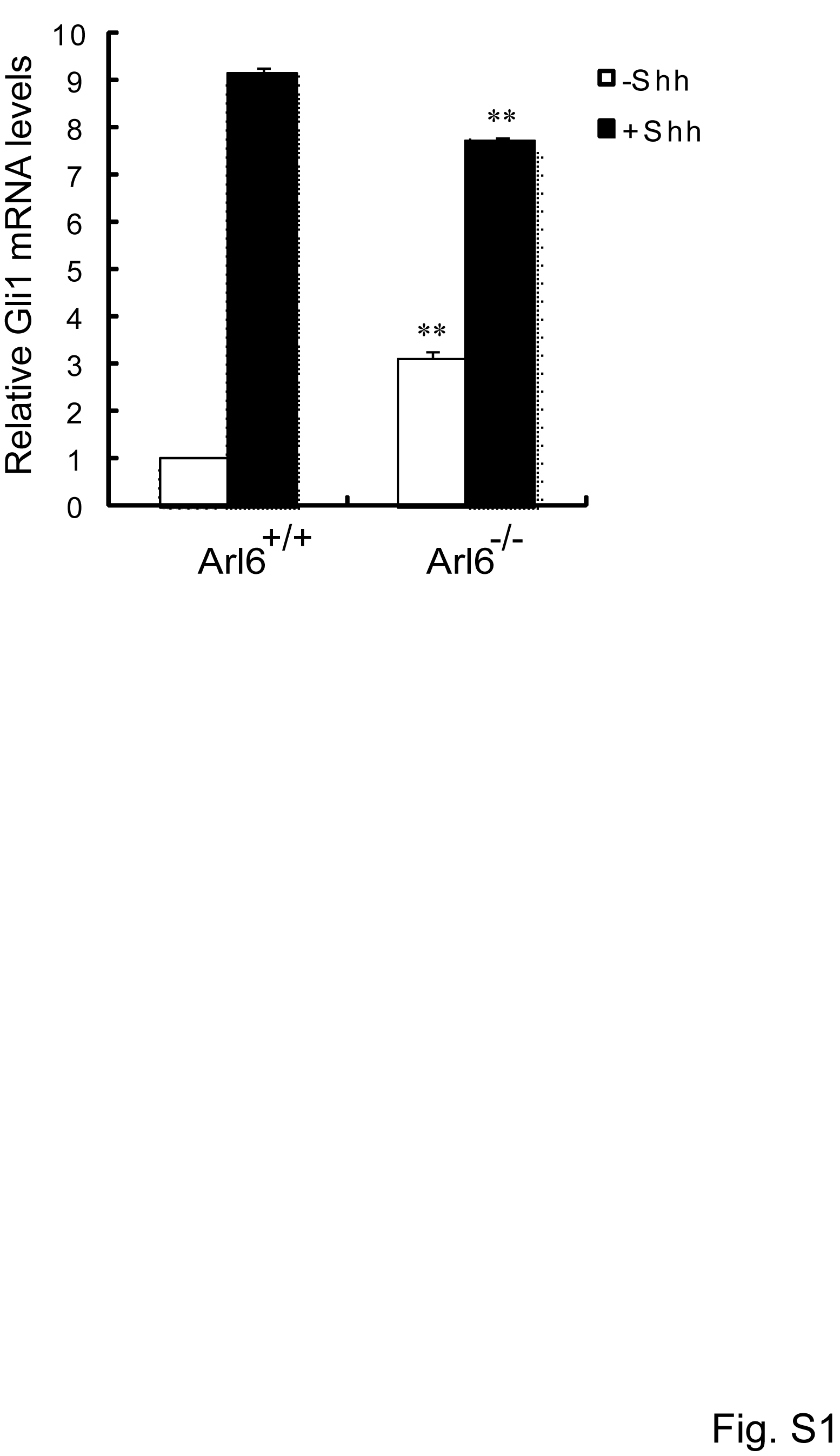

Supplement: Supplementary file 1 — Additional file 1: Figure S1. Knockout of Arl6 reduced Hedgehog signaling activity in MEFs. Detection of Gli1 in Arl6 knockout MEFs by RT-PCR (Fig. 1e). The relative intensity of the bands is shown by semi-quantified analysis with the software Image J. The results represent the mean ± SEM of three independent experiments. **P < 0.01 compared with Arl6+/+. [file 13578_2016_126_MOESM1_ESM.tif]

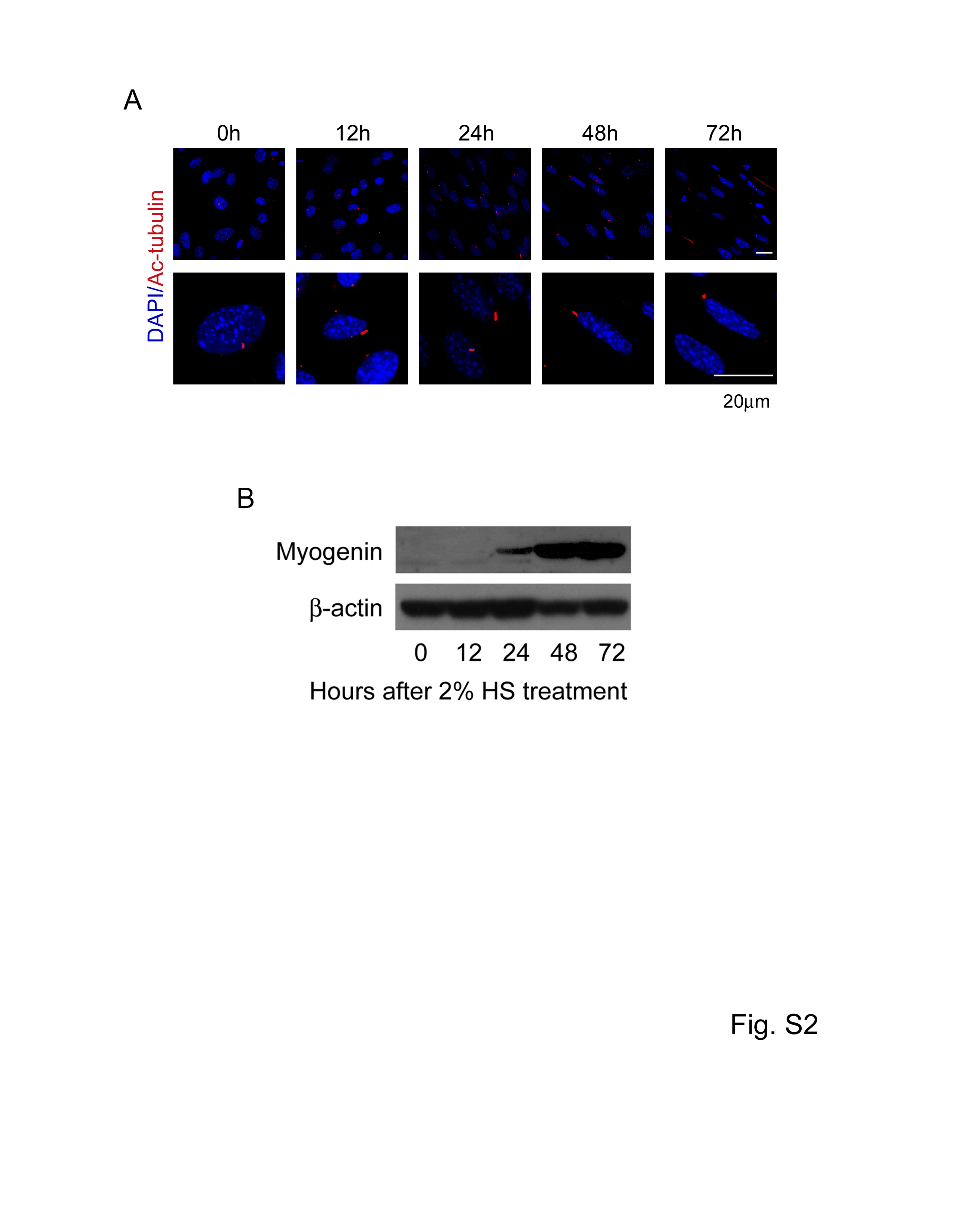

Supplement: Supplementary file 2 — Additional file 2: Figure S2. Cilia assemble and dissemble in myoblast differentiation. (A) Representative confocal images showing the anti-acetylated tubulin (green) staining of primary cilia on C2C12 during differentiation. (B) Western blot analysis of Myogenin levels during differentiation. β-actin was used as a loading control. [file 13578_2016_126_MOESM2_ESM.tif]

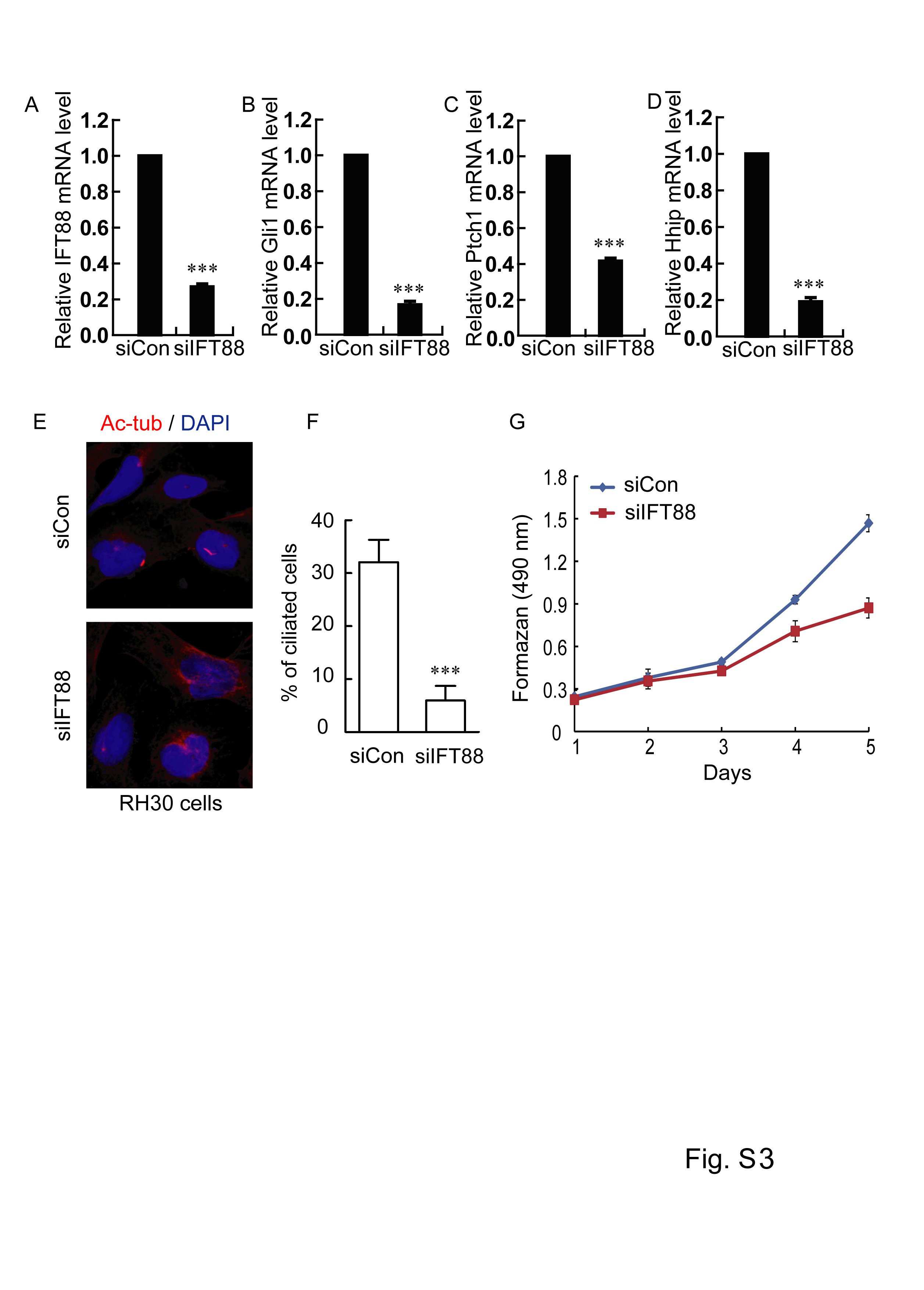

Supplement: Supplementary file 3 — Additional file 3: Figure S3. Knockdown of IFT88 suppresses cell growth of RH30. (A) The knockdown efficiency of IFT88 siRNA in RH30 was detected by Q-PCR. (B-D) Q-PCR detection of Gli1, Ptch1 and Hhip mRNA levels in RH30 cells knockdown of IFT88. (E) Representative confocal images and (F) percentage quantitation of primary cilia in RH30 cells expressing siIFT88 or siCon. (G) MTT assays for RH30 cells expressing siIFT88 or siCon. The results represent the mean ± SEM of three independent experiments. ***P < 0.001 vs. siCon. [file 13578_2016_126_MOESM3_ESM.tif]

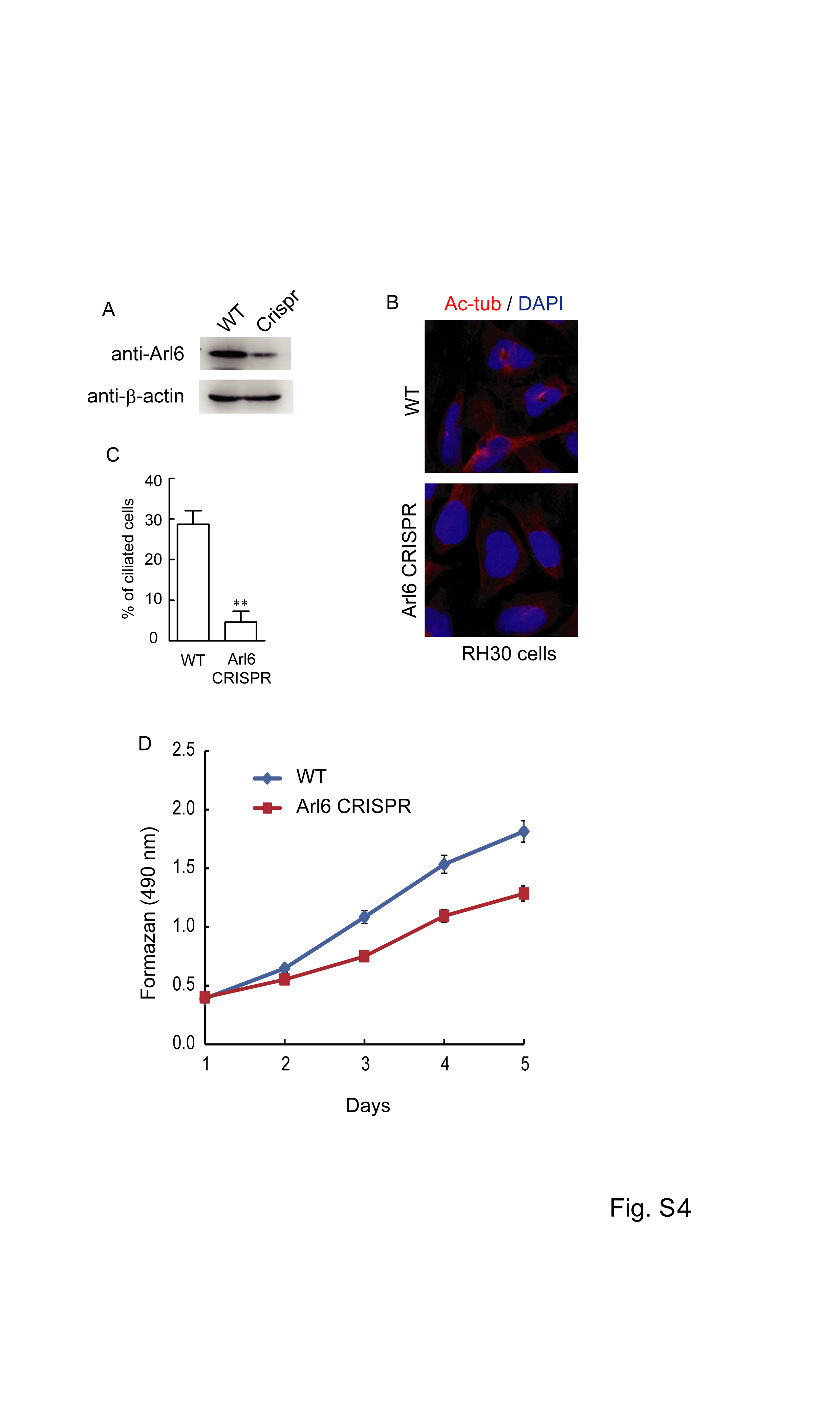

Supplement: Supplementary file 4 — Additional file 4: Figure S4. Arl6 CRISPR suppresses cell growth of RH30. (A) Western blot evaluated Arl6 protein levels in selected individual RH30 clones edited by Arl6 CRISPR. (B) Representative confocal images and (C) percentage quantitation of primary cilia in RH30 edited cells. The results represent the mean ± SEM of three independent experiments. **P < 0.01 vs. WT (D) MTT assays for RH30 edited cells. [file 13578_2016_126_MOESM4_ESM.tif]
